# The epidemic of the multiresistant dermatophyte *Trichophyton indotineae* has reached China

**DOI:** 10.3389/fimmu.2022.1113065

**Published:** 2023-02-16

**Authors:** Songgan Jia, Xuemei Long, Wei Hu, Jiali Zhu, Yinhui Jiang, Sarah Ahmed, G. Sybren de Hoog, Weida Liu, Yanping Jiang

**Affiliations:** ^1^Department of Dermatology, The Affiliated Hospital of Guizhou Medical University, Guiyang, China; ^2^Department of Microbiology, Basic Medical School, Guizhou Medical University, Guiyang, China; ^3^Laboratory of Medical Molecular Biology, Guizhou Medical University, Guiyang, China; ^4^Centre of Expertise in Mycology, Radboud University Medical Center/Canisius Wilhelmina Hospital, Nijmegen, Netherlands; ^5^Department of Medical Mycology, Institute of Dermatology, Chinese Academy of Medical Science & Peking Union Medical College, Nanjing, China; ^6^Center for Global Health, School of Public Health, Nanjing Medical University, Nanjing, China; ^7^Infectious Dermatology Group, Jiangsu Key Laboratory of Molecular Biology of Skin and STIs, Nanjing, Jiangsu, China

**Keywords:** arthrodermataceae, recombination, genotype, natural resistance, China

## Abstract

Due to its high degree of natural resistance to terbinafine *in vitro* and its tendency to spread globally from the Indian subcontinent, the emerging dermatophyte *Trichophyton indotineae* has become a major concern in dermatology. Herein, we present the first report of *T. indotineae* from mainland China. The transmission of the fungus to Guizhou Province in central China and eventual host susceptibilities were investigated. We studied 31 strains of the *T. mentagrophytes* complex from outpatient clinics of our hospital collected during the past 5 years. The set comprised four ITS genotypes, two of which were *T. mentagrophytes* genotype VIII, now known as *Trichophyton indotineae*; the earliest isolation in the Guiyang area appeared to date back to 2018. The isolate was derived from an Indian patient, while local Chinese patients had no dermatophytosis caused by this genotype. Reports from around the world indicated that almost all of the globally reported *T. indotineae* cases originated from the Indian subcontinent and surrounding countries without transmission among native populations, suggesting deviating local conditions or racial differences in immunity against this fungus.

## Introduction

Dermatophytes are among the most common fungal pathogens of animals and humans worldwide (1), affecting up to 20-25% of the world’s population (2). Traditionally, dermatophytosis is not a life-threatening infection and its health impact can be relatively low, and thus it represents a rather neglected infection in many regions. It is of concern that an increasing number of difficult-to-treat and invasive dermatophytosis have been reported in the last two decades (3), especially in Patients with CARD9 or STAT3 mutations where a mortality rate as high as 17.4% has been noted (4). More attention has been drawn in the last five years by the emergence of terbinafine (TBF) -resistant population in the *Trichophyton mentagrophytes* complex in India (2, 5, 6). The emerging pathogen has been described as the novel species *T. indotineae* (7), a distinct clonal offshoot in the *T. mentagrophytes* complex (8), formerly described as *T. mentagrophytes* rDNA ITS genotype VIII in 2019 (9–11). While dermatophytes in general are susceptible to most commonly used antifungals, *T. indotineae* is resistant and was alaming in the speed of its spread on the Indian subcontinent, replacing *Trichophyton rubrum* as the dominant dermatophyte (10, 12).

Analysing the geospatial emergence of *T. indotineae* (*T. mentagrophytes* genotype VIII) lends useful insights into the potential factors that could have driven its origin and spread. Interestingly, *T. mentagrophytes* genotype VIII was not originally found in India: the earliest isolate was collected in Australia in 2008 (12, 13) and subsequently it was isolated in Oman (2010) and Iran (2016) (14). Confusion arose because these earliest collected strains adopted a different species name, *T. interdigitale*, based on an older taxonomic standard of dermatophytes which was valid in 2016 (10). The period around 2017-2018 seemed to have been a watershed, after which this unique multidrug-resistant clonal offshoot rapidly emerged in India (10, 15, 16). A similar emergence was noted concomitantly in Iran (14, 17), leading to frequent and recurrent tinea corporis, tinea cruris, tinea faciei and their combinations. Although the severity of inflammation varies, the itching is intense (10, 18). With migration and travel, the anthropophilic propagules gradually spread from the Indian continent to Europe and the Americas, starting in 2018 in Germany (10, 16), in 2020 in Japan (19), in 2021 in Belgium, Switzerland, Greece (20–22), in 2022 in Turkey, France, Denmark, Australia, Canada and most recently in Vietnam (23–29). The dermatophytosis caused by *T. indotineae* has now been reported in many countries around the world.

China, with its large population in Asia and bordering India, so far did not reported any cases of *T. indotineae.* Recently we successfully treated an Indian patient with tinea corporis, tinea cruris and tinea faciei caused by *T. indotineae* in our dermatology clinic. To the best of our knowledge, this is the first report of this species from mainland China. Therefore, the primary aims of this study are (i) to investigate the potential transmission and epidemics of the *T. mentagrophyte*s complex in the wider Guiyang area, and (ii) to further understand the biological behavior and host factors impacting *T. indotineae*.

## Materials and methods

### Strain and specimen collection

The clinical samples were collected from patients who visited the dermatology clinic of the Affiliated Hospital of Guizhou Medical University, Guiyang, China, between May 2017 and April 2022. Identification of all dermatophytes except *T. rubrum* was confirmed by internal transcribed spacer (ITS) rDNA sequencing. A total of 31 isolates of the *T. mentagrophytes* complex were isolated and examined. The information on source of isolation and other metadata can be found in **Table S1**. Nail fragments, hair and skin scales of the patients were collected and divided for cultivation and direct microscopy (with/without Calcofluor in 10% potassium hydroxide). The untreated samples were inoculated on the slope of Sabouraud’s Glucose Agar (SGA; homemade) containing 100 mg/L chloramphenicol and cultured at 28°C for two weeks. The strains with good growth, successful isolation and purification, and morphological identification as dermatophytes were included in the study.

### Phenotype and preservation

Isolates of *T. indotineae* (JYP18010 and JYP22048) were grown on various agar media, including SGA and potato dextrose agar (PDA; HiMedia) at 30°C. Spore suspension of strains JYP22048 and JYP18010 (10^6^ CFU/mL) were obtained, and 1μL (10^3^ CFU) suspension was inoculated in the center of a PDA plate (9 cm). Morphology and growth rates of colonies were determined during two weeks in the dark at temperatures of 4, 18, 25, 30, 37 and 40°C. The colonies were documented using a Canon EOS 500D camera. Micromorphology was observed and documented. Production of urease was determined in Christensen’s urea (HuanKai Microbial, China) broth after incubation at 30°C for 3 and 7 days, with a final check after 10 days. Orange and pink tubes were scored as negative and positive, respectively. Strain *T. mentagrophytes* JYP18049 (=JYP18-3) was used as positive control (30). All strains were sequenced and stored in 15% glycerol in a refrigerator at -80°C.

### Identification and phylogeny

Identification of isolates was done by phenotype (31) and confirmed by rDNA ITS sequencing. Briefly, isolates were subcultured on SGA (homemade) and incubated at 28°C for one week. DNA extraction was by the cetyltrimethylammonium bromide (CTAB) method (32). ITS of the rDNA operon was amplified with primers ITS1 and ITS4 according to Jiang et al. (32, 33). PCR products were sequenced by TSINGKE Biological Technology (Beijing, China). Sequencing results were assembled by SeqMan (DNASTAR), and were compared by BLAST in GenBank. A total of 31 isolates of the *T. mentagrophytes* complex were isolated and examined in this study. GenBank accession numbers for new sequences are given in **Supplementary Table S1**. For global comparison, 115 reference sequences were retrieved from GenBank, including *Trichophyton mentagrophytes* complex (n=108), *T. schoenleinii* (n=3), *T. tonsurans* (n=1), *T. equinum* (n=1), and with *T. simii* (n=2) as outgroup. These include the neotype and type strains of *Trichophyton* species (*T. interdigitale*, CBS 428.63^NT^; *T. mentagrophytes*, IHEM 4268^NT^; *T. indotineae*, CBS 146623^T^; *T. equinum* CBS 100080^T^; *T. tonsurans*, CBS 338.37^NT^; *T. simi*, CBS 449.65^T^). Reference strains for genotypes of *T. mentagrophytes* (ITS genotypes III, III^*^, IV, VII, VIII, XI) and *T. interdigitale* (ITS genotypes I, II) were included in order to precisely reproduce the population structure (9, 10). Alignment was done with MAFFT v7 (https://mafft.cbrc.jp/), then analyzed using maximum-likelihood (ML) and Bayesian inference (BI) methods. Suitable substitution models of ML trees were determined using MEGA v6.0. ML in the CIPRES web server (https://www.phylo.org). Bayesian posterior probabilities were calculated using MRBAYES v3.2.7 (34). Phylograms are shown using FIGTREE v1.3.1 (35). Bootstrap values ≥ 80% and posterior probabilities ≥ 0.97 were considered as statistically supported and were indicated above thickened branches.

### Antifungal susceptibility

Antifungal susceptibility testing was performed for isolates JYP18010 and JYP 22048 using the Clinical and Laboratory Standards Institute (CLSI) M38-A2 broth Dilution Antifungal Susceptibility Testing of Filamentous Fungi (36). MICs were determined after 24 h of incubation at 35°C. *Candida parapsilosis* ATCC 22019 and *Trichophyton mentagrophytes* ATCC MYA-4439 were used as quality controls. Terbinafine (TBF; CFDA Co., Beijing, China), luliconazole (LLCZ; Higher Biotech Co, Shanghai, China), itraconazole (ITZ; CFDA), voriconazole (VCZ), fluconazole (FCZ; Sigma Aldrich), ketoconazole (KTZ; CFDA) were applied. Stock solutions of all drugs were prepared in dimethyl sulfoxide (DMSO) at a concentration of 1600 µg/ml (except FCZ, which was dissolved in distilled water at final concentration 6400 µg/ml).

### Squalene epoxidase gene

The isolates of *T. indotineae* (JYP18018 and JYP22048) were screened for mutations in the squalene epoxidase (*SQLE*) gene. DNA was amplified using primers SQLE3S (5’-GTGTAAAGGGTCACATGCGG) and SQLE4R-2 (5’-AAGTTCGGCAAATACGAAAG) (7). PCR amplification (T100 Thermal Cycler; BIO-RAD, America) was carried out for 30 cycles consisting of denaturation for 30 sec at 95°C (Preheating for 5 min at 95°C), annealing for 30 sec at 55°C and extension for 30 sec at 72°C. A final extension was performed at 72°C for 5 min. The amino acid sequences of *SQLE* of the two *T. indotineae* isolates in the present study were compared with the reference sequence of *T. mentagrophytes SQLE* (TIMM 2789, GenBank accession no. KU242352).

### Literature search

We performed a search of published cases/GenBank accession no. of *T. mentagrophytes* VIII or *T. indotineae* in PubMed and Embase using the keywords “dermatophytoses”, “terbinafine resistance”, “*Trichophyton. indotineae*”, “*T. mentagrophytes* Genotype VIII” and “ITS region”. We only reviewed cases with clinical information and laboratory/sequence data to identify the fungus as *T. mentagrophytes* genotype VIII or *T. indotineae*, for cases and GenBank accession numbers of strains reported from India and Iran is referred to literature. Data recorded included case year, race where known, and country of origin.

## Results

### Tinea corporis/tinea cruris due to T. indotineae

**Figure 1:**
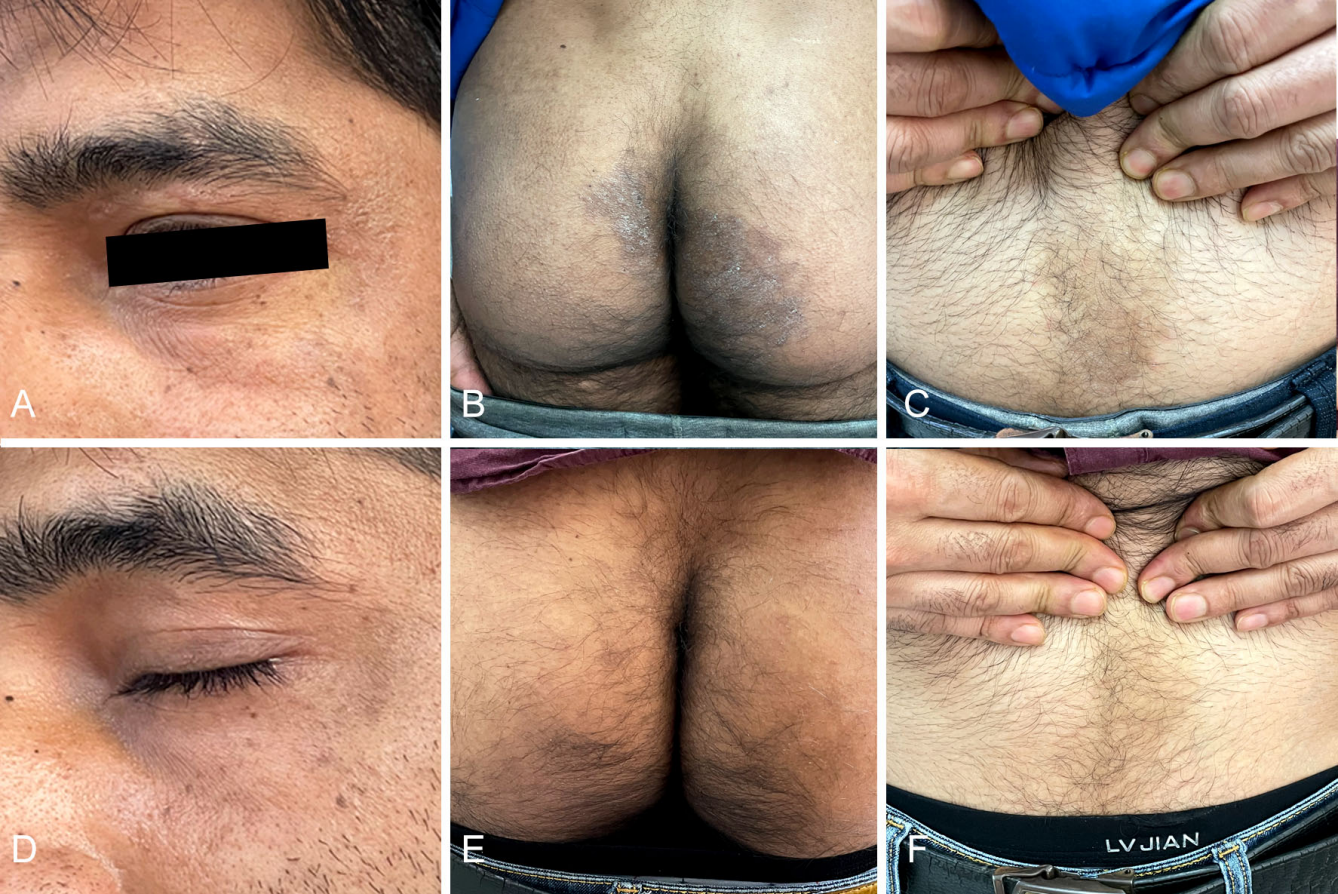
Irregular dark erythema ranging from coin to palm size with a small amount of fine furfur-like desquamate on the surface **(A, B, C)**; Four weeks after treatment with itraconazole **(D, E, F)**.

**Figure 2:**
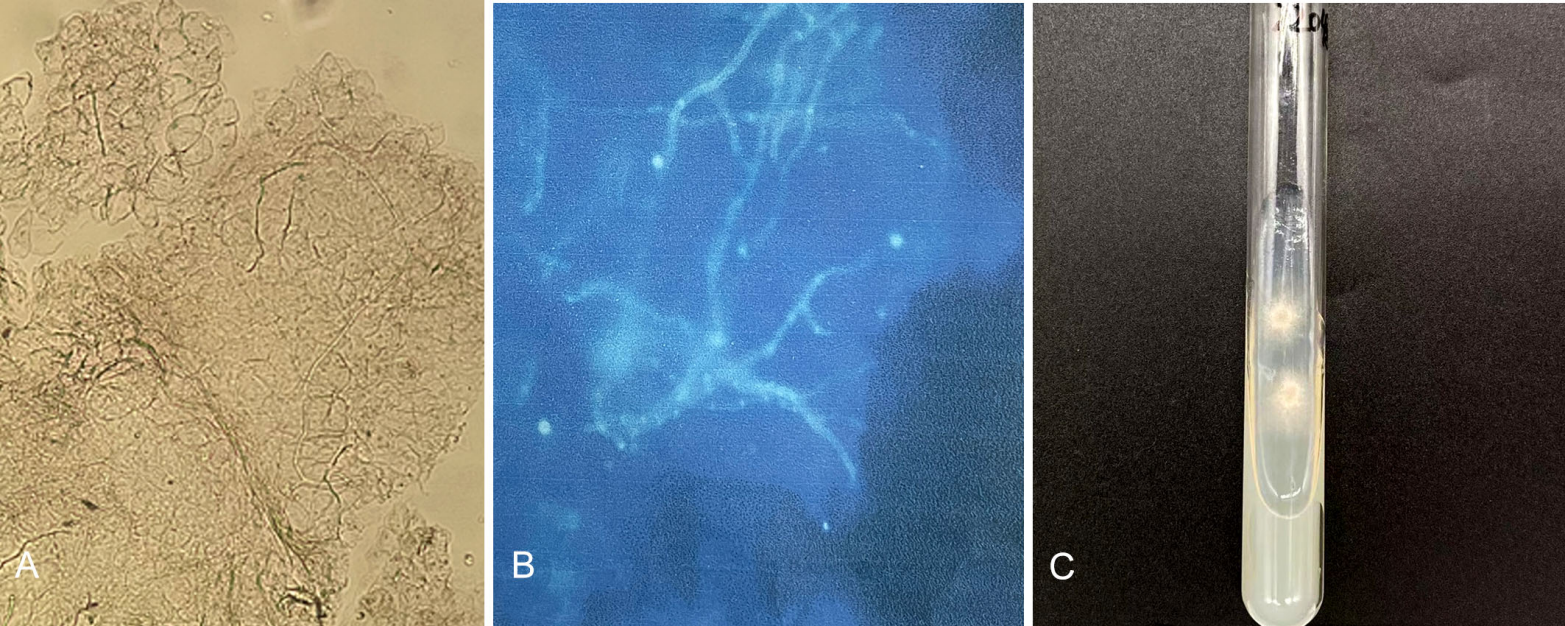
Direct examination (KOH 10% and Blankophor) showed disarticulating hyphal elements **(A, B)**; One velvety isolate (JYP22048) was obtained from the eyebrow sample on SDA two weeks at 28°C **(C)**.

A 32-year-old Indian male patient presented at the Dermatology clinic of our hospital in March 2022 with a 6-month history of pruritic eruptions. The rash started on the buttocks and then spread to the abdomen and left eyebrow, presenting as irregular dark erythema ranging from coin to palm size, with a small amount of fine furfur-like desquamation on the surface (**Figures 1A–C**). He has lived in Guiyang for four years and had not traveled back to India or elsewhere during past four years. There was no history of animal contact or of contact with similar patients, but his father in India also developed rashes and itching for nearly one year. The patient was initially diagnosed with tinea corporis and tinea cruris in another hospital four months earlier. Fluconazole 50 mg once a day was taken orally for two weeks, and miconazole nitrate cream was applied locally for 4 months, but there was no visible improvement. Direct examination (KOH 10% and Blankophor) showed septate, bright hyphal elements (**Figures 2A, B**) in samples from scales of the buttocks, abdomen and left eyebrow. Cultures from lesions of the patient were performed on SGA incubated for two weeks at 28°C. Only one velvety isolate (JYP22048) (**Figure 2C**) was obtained from the eyebrow sample and was preliminarily identified based on morphological features as a zoophilic strain of *Trichophyton interdigitale* according to the concept of Heidemann et al. (37). The identification was subsequently corrected to *T. mentagrophytes* type VIII after ITS rDNA sequencing and phylogenetic analysis. Oral treatment with itraconazole (200 mg daily) in combination with bifonazole (1% cream, two times a day) and ketoconazole (2% lotion, once a day) was initiated based on the mycological result. To prevent anaphylaxis, oral glycyrrhizin tablets (100 mg/d p.o. every 12 h) was given for 2 weeks. Following this approach, remission was observed, and the lesions became smaller and without scaling. A cure was achieved 4 weeks after starting the treatment, with only pigmentation remaining (**Figures 1D–F**). No recurrence has been observed to date.

The second case concerned a 20-year-old Indian girl who presented to our outpatient clinic in July 2018 with rash and pruritus at the root of both thighs. The patient, an international student of Guizhou Medical University, had been in Guiyang for 3 years before onset of illness, during which she had not returned to India and had no contact history. Tinea cruris was diagnosed after positive microscopic examination of skin scales. Itraconazole (200 mg orally daily) was given for 1 week and the disease was relieved. There was no recurrence in the telephone follow-up. Clinical and microscopic images were not collected, but positive strain isolation was achieved from a skin scale of the patient and referred to as JYP18010; this strain was confirmed to be *T. mentagrophytes* genotype VIII by ITS sequencing. The source of the infection was not found.

### Tinea cases due to T. mentagrophytes complex

In total, 30 cases of dermatophytosis were caused by members of the *T. mentagrophytes* complex. Strains were identified during retrospective analysis of the rDNA internal transcribed spacer generated during epidemiological surveys in the Affiliated Hospital of Guizhou Medical University between 2017 and 2022. The cases are summarized in **Table 1**. Combined with molecular sequencing and phylogenetic analysis in this study, 30 patients (31 strains) belonged to four ITS genotypes of the *T. mentagrophytes* complex, including *T. interdigitale* types I and II (16 cases), *T. mentagrophytes* type VII (3 cases), *T. mentagrophytes* type IX (9 cases) and *T. mentagrophytes* type VIII (2 cases). Except for the two hosts of *T. mentagrophytes* type VIII, which were Indian, all other patients were Chinese nationals from Guizhou.

**Table 1:** Demographic and clinical data of patients with *T. mentagrophyte*s/*T. interdigitale* complex (TMTISC) by ITS genotypes.

Basic information		*T. mentagrophytes* IX	*T. mentagrophytes* VII	*T. mentagrophytes* VIII	*T. interdigitale* I/II
**Gender**	male	4	2	1	7
	female	5	1	1	9
	total	9	3	2	16
**Age**	average(min-max)	26(3-73)	25(7-35)	27(22-32)	34(4-66)
	unknown	1	0	0	1
**Animal contact history**	yes	3	3	0	4
	no	4	0	2	9
	unknown	2	0	0	3
**Patient contact history**	yes	1	3	0	3
	no	6	0	2	10
	unknown	2	0	0	3
**Dermatophytosis**	Tinea capitis	4	0	0	1
	Tinea faciei	1	0	0	5
	Tinea corporis	0	2	0	0
	Onychomycosis	2	0	0	8
	Tinea inguinalis	1	0	1	0
	Multiple areas	0	1	1	2
	unknown	1	0	0	0
**Inflammation**	mild	4	2	1	10
	severe	3	1	1	3
	unknown	2	0	0	3
**Duration of illness before visiting clinic**	average(week)	100	23	3	83
	unknown	2	0	0	3

*Trichophyton interdigitale* types I and II patients were mostly adults, with an average age of 34 years (range 4–66 years, with 5 children under 8 years old). The infection sites of 5 pediatric patients were all scalp (1 case) and face (4 cases). Patient with isolate JYP21091 was an 8-year-old child with a history of contact with cats and rabbits, which resulted in severe inflammation of tinea capitis, and a scalp scar and permanent hair loss were left after 6 months of treatment. The main site of infection in 11 adult cases was nail (9/11, 82%), in addition to one causing tinea corporis and tinea cruris with severe inflammation (strain JYP21100), and one causing tinea faciei in a 44-year-old woman (strain JYP21071). Nine cases of *T. mentagrophytes* type IX were mainly children under the age of eight (5/9, 55.6%), presenting with tinea capitis and tinea faciei. In one case, a 73-year-old male (strain JYP18108) presented with an infected toenail. *Trichophyton mentagrophytes* type VII (3 cases) concerned a family of three previously reported (30), with a history of close contact with rabbits.

### Phylogeny

**Figure 3:**
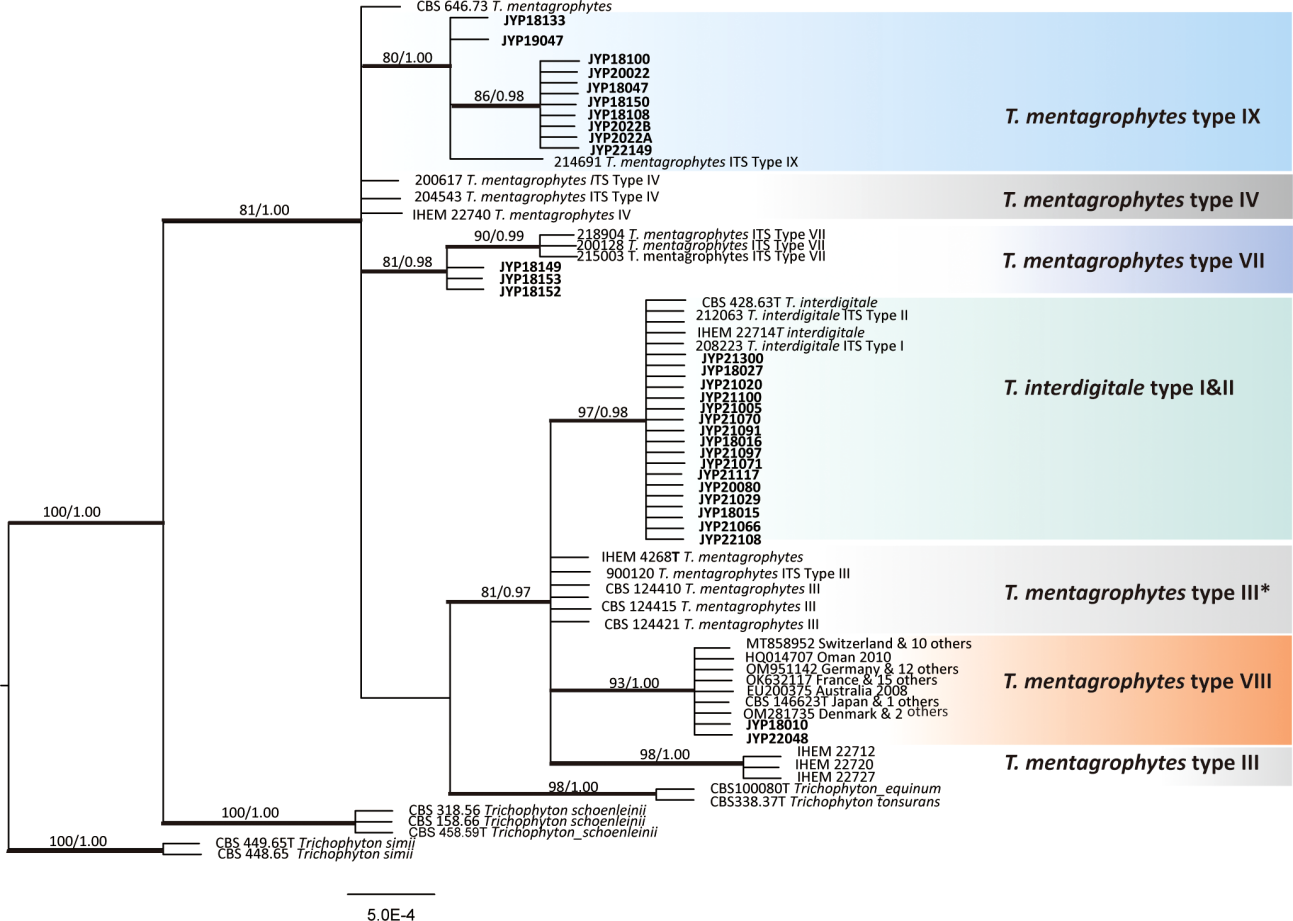
Phylogenetic tree constructed through Bayesian inference analyses based on ITS sequences showing relationships of eight genotype belonging to the *T. mentagrophyte*s/*T. interdigitale* complex (TMTISC). Only support values exceeding bootstrap values of 70% and Bayesian posterior probabilities of 0.95, respectively, are shown. Ex-type isolates are designated by a superscript T. *Trichophyton simi* CBS 449.65 was used as the outgroup. Guizhou strains are highlighted in bold.

**Figure 4:**
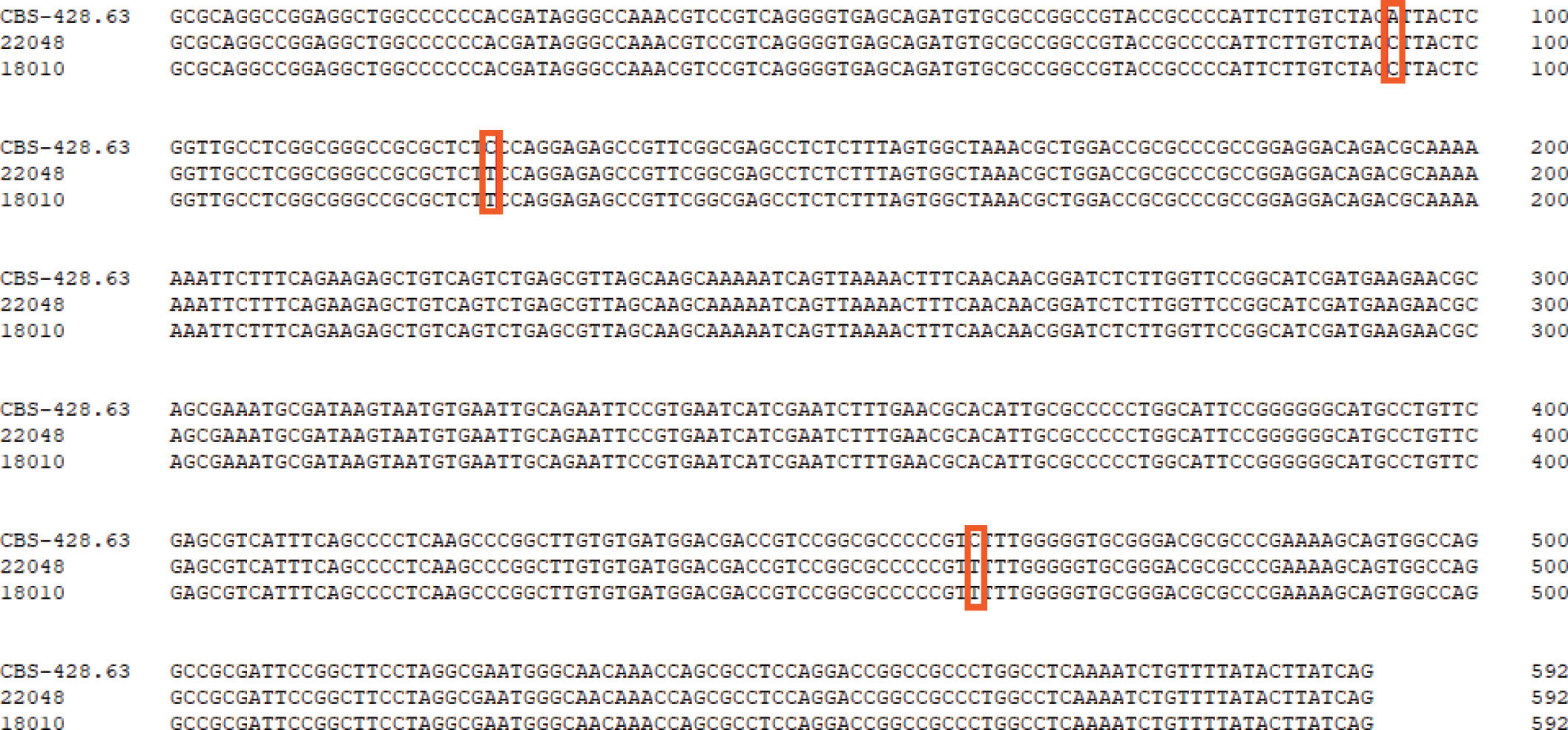
Comparison of the ITS regions of the nucleotide sequences of the isolate of JYP22048, JYP22010 and *T. interdigitale* CBS 428.63 (NR_144900). Boxes indicate specific SNP between high TRF-resistance strains and the reference strains of *T. interdigitale*.

For phylogenetic analysis, we used 115 previously published ITS sequences from members of the *T. mentagrophytes* complex, as well as the type strains of *T. tonsurans* and *T. equinum* that were previously confirmed to branch within the clade (38). The phylogenetic analysis indicated that the group of *T. mentagrophytes* complex, *T. tonsurans*, and *T. equinum* formed a single terminal cluster without prominent branches with strong bootstrap support (ML/BI 100/1.00). *Trichophyton schoenleinii* was placed in a basal position to this cluster with a Bayesian posterior probability of 1.00 (**Figure 3**). The 31 strains in this study were scattered in four subclades with strong bootstrap support, 16 strains clustered in ITS type I and II subclade (ML/BI 97/0.98), 10 strains clustered in ITS type IX subclade (ML/BI 80/1.00), 3 strains in ITS type VII subclade (ML/BI 81/0.98) and 2 strains in ITS type VIII subclade (ML/BI 93/1.00). So far, no strains collected from Chinese Guizhou patients were found to cluster in the ITS type VIII subclade, which has been described as *T. indotineae*. Compared to CBS 428.63 (neotype of *T. interdigitale*), our two isolates (JYP18010 and JYP22048) harbored three single polymorphisms (SNPs) at position 94 (C), 125 (T) and 462 (T), identical to the remaining strains located in the subclade (**Figure 4**), whereas they differed from IHEM 4268 (neotype of *T. mentagrophytes*) in 2 positions in ITS.

It should be noted that our data did not allow to resolve the delimitation of *T. mentagrophytes* and *T. interdigitale*, since genotypes I and II representing *T. interdigitale* and III, III^*^, VII, IX, and VIII representing *T. mentagrophytes* were all mixed together and did not form monophyletic clades, respectively. Moreover, the neotype strains of *T. mentagrophytes* (IHEM 4268), *T. interdigitale* (CBS 428.63) and *T. mentagrophytes* type VIII (*T. indotineae* CBS 146623) were located in the same clade with strong Bayesian posterior probability (ML/BI, 81/0.97). The relationships of these three species with other taxa (*T. tonsurans* and *T. equinum*) in the *T. mentagrophytes* clade are poorly resolved due to insufficient statistical support. Consequently, the level of intraspecific variability, along with the precise numbers of species-specific substitutions or indels useful for their differentiation, needs to be confirmed in future studies when more strains will be available.

### Physiology and morphology

**Figure 5:**
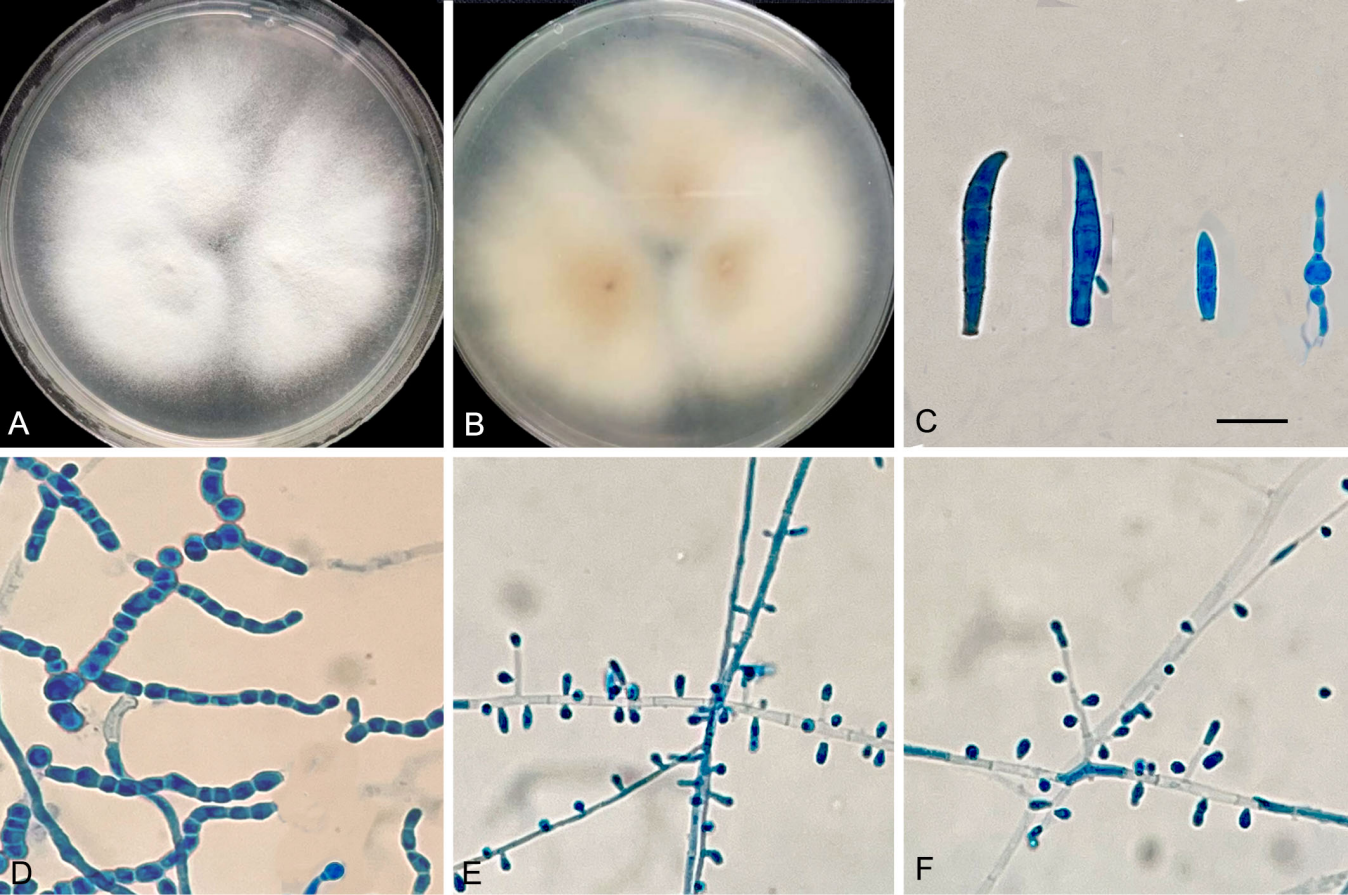
*T. mentagrophytes* genotype VIII (JYP 22048). Colonies on PDA 7 days at 30°C **(A, B)**; Macroconidia **(C)**; Chlamydoconidia **(D)**; Subspherical to pyriform microconidia **(E, F)**; Scale bars: 10μm.

**Figure 6:**
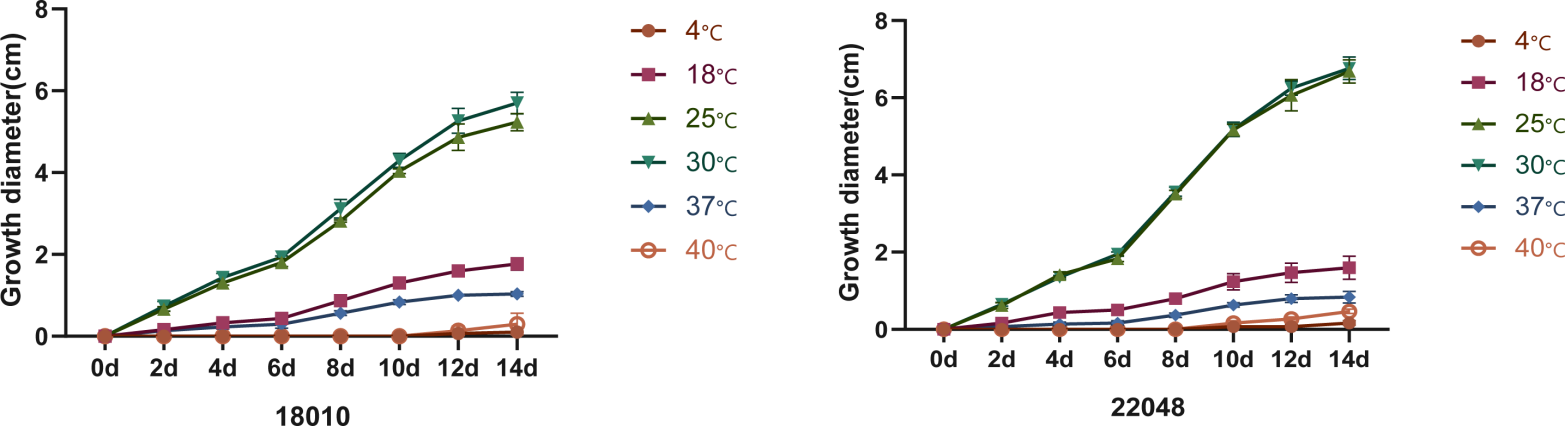
Temperature curves of strains JYP18010 and JYP22048 growing on PDA.

Macromorphology of *T. mentagrophytes* type VIII (*T. indotineae*) is shown in **Figure 5**. Colonies of the isolates (JYP18010 and JYP22048) were flat, white to cream in color, with a velvety surface, and light orange yellow reverse with no diffusible pigment on PDA at 30°C for 2 weeks (**Figures 5A, B**). Numerous subspherical to pyriform microconidia were present and spiral hyphae were absent (**Figures 5E, F**). Macroconidia were few and cigar- to club-shaped, with three to five septa, and were smooth and thin-walled with narrow attachment bases (**Figure 5C**). Isolate JYP22048 demonstrated beaded chains of chlamydoconidia on PDA at 30°C (**Figure 5D**). The optimum growth temperature of the two strains was 25–30°C on PDA reaching a colony diameter of 70 mm in 14 days, 7 mm diameter at 37°C, and slow growth at 40°C for JYP22048, without growth at 40°C for JYP18010; minimum growth temperature 4°C (**Figure 6**). Absence of urea hydrolysis was observed in the two strains of *T. indotineae* (JYP18010 and JYP 22048) after incubation for 10 d at 30°C.

### Antifungal susceptibility

**Figure 7:**
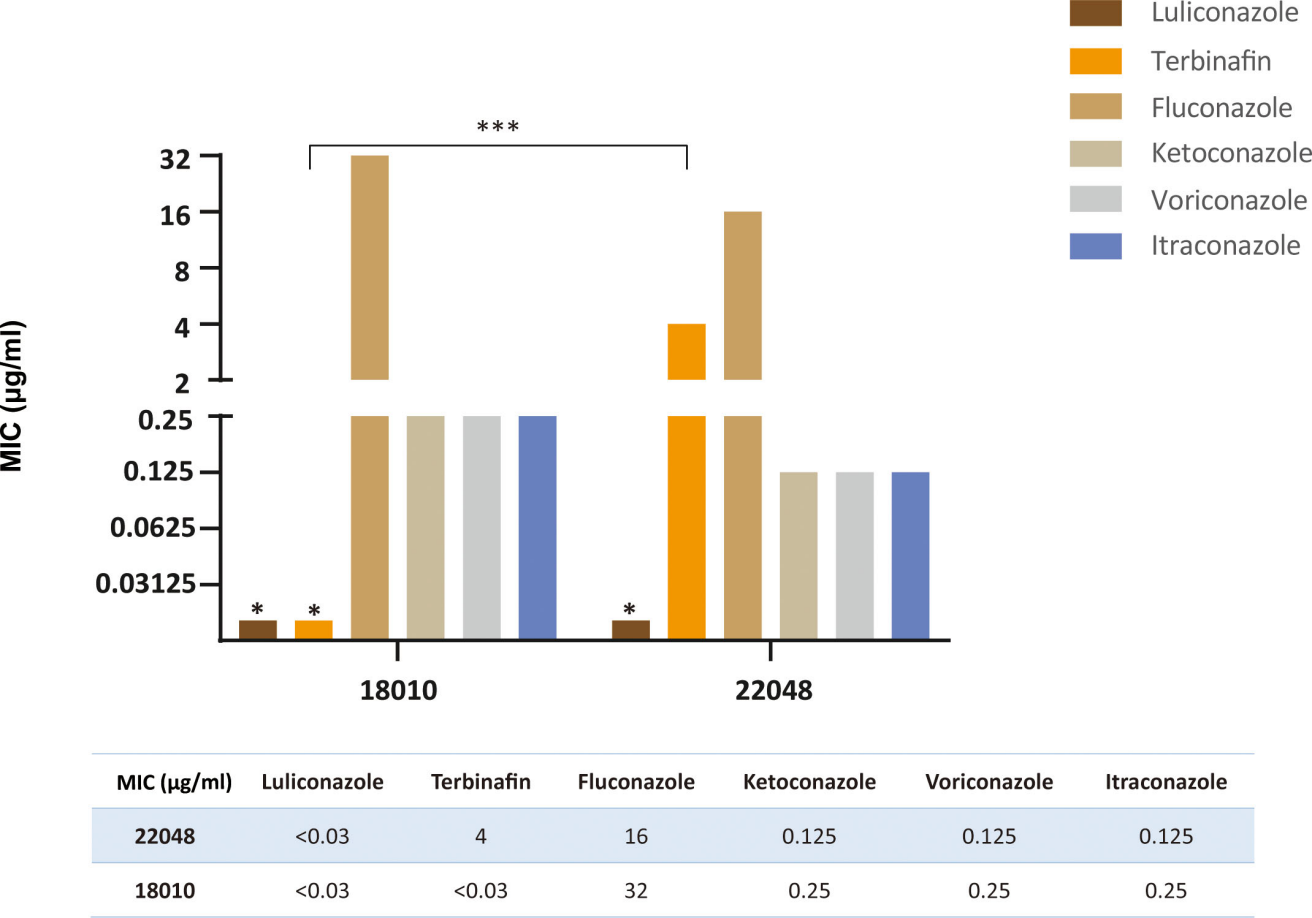
Comparison of MIC values of two strains (JYP22048 and JYP18010) to six antifungal agents. (*** means P<0.01, * means MIC<0.03 mg/L).

**Figure 7** compares the MIC values of 6 antifungal drugs against the two clinical isolates of *T. indotineae*. Except for TBF, the remaining drugs showed similar antifungal activities against JYP18010 and JYP22048. Isolate JYP22048 had a high MIC value (4 µg/ml) against TBF, whereas isolate JYP18010 had a low MIC of < 0.03 µg/ml. Among triazoles, FCZ showed reduced susceptibility against *T. mentagrophytes* type VIII strains and the two isolates proved to be resistant, with MIC values of 16 µg/ml and 32 µg/ml, respectively. LLCZ was the most active antifungal drug against the two strains, with lowest MIC (< 0.03 µg/ml). KTZ showed good antifungal activity, similar to VCZ and ITZ, with MIC values of 0.25 µg/ml (JYP18010) and 0.125 µg/ml (JYP22048), respectively.

### The mutation hotspot (Phe^397^Leu) of SQLE

The partial *SQLE* sequence was successfully amplified in our two *T. mentagrophytes* type VIII strains (JYP18010 and JYP22048). The TBF-resistant strain (JYP22048) encoded Leu at codon 397 instead of Phe (Phe^397^Leu) in *T. mentagrophytes* strain TIMM2789 (GenBank accession number KU242352). The susceptible isolate JYP18010 showed no mutation. The *SQLE* sequence has been deposited in the GenBank database under accession number OQ054983 and OQ054984.

### Review of published cases

**Figure 8:**
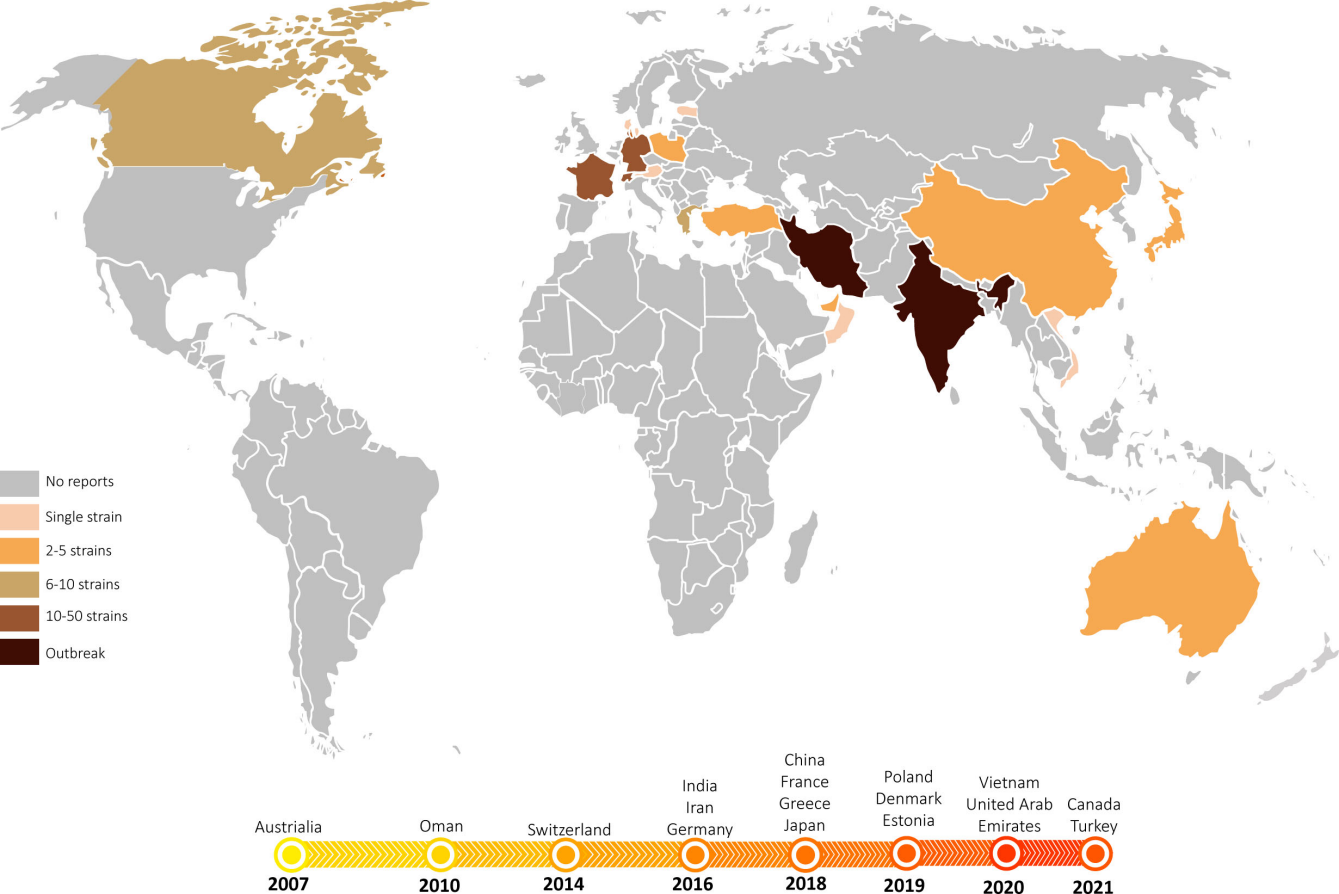
The emergence and spread of *T. indotineae*. World map showing the countries where *T. indotineae* has been isolated to date. Most countries have detected multiple cases in more than one healthcare institution, with some countries experiencing prolonged outbreaks. In contrast, some countries have so far reported only single cases with no further transmission. The timeline below depicts the years in which *T. mentagrophytes* type VIII was first isolated in different countries, showing near-simultaneous emergence and spread of *T. indotineae* across the Indian continent to Europe and the Americas between 2008 and 2022.

We reviewed 15 articles containing 100 cases or sequences (including the current study) of *T. mentagrophyte*s type VIII*/T. indotineae* infection published in the period 2008−2022 (**Table 2**, **Figure 8**). In addition to India and Iran, 15 countries have reported cases or collected strains of this genotype so far. A total of 80 (80%) cases were reported from Europe, two from Australia, ten (10%) from Asia and eight from U.S.A., respectively, while no case was from Africa. However, the host origin of cases or strains found locally in these countries was still India (35 cases, 35%), followed by Bangladesh (11 cases, 11%); The remaining 29 cases were of no race or nationality. Strain collection time, patient race and strain numbers or GenBank accession numbers are shown in **Table 2**.

**Table 2:** Basic information on hosts of *T. indotineae* strains collected in other countries except India and Iran.

Data of publish	Country	Culture accession No.	Collection Time	GenBank Accession Number (ITS)	Origin	Reference
2022	China	JYP18010	2018	OP961393	India	Our study
		JYP22048	2022	OP961421	India	
2022	Turkey	CBS 149165/1128	2021	ON528186	Turkey	Durdu M, et al. (23)
		CBS 149166/1129	2021	ON528187	Turkey	
2022	France	3	2019	MW959755	Bangladesh	Jabet A, et al. (24)
		9	2021	MW959756	Bangladesh	
		5	2020	MW959757	Bangladesh	
		6	2020	MW959758	Myanmar	
		7	2020	MW959759	Bangladesh	
		10	2021	MW959760	Bangladesh	
2022	France	CR-1	2018	MW898018	Bangladesh	Dellière S, et al. (25)
		CR-2	2018	MW898019	India	
		CR-3	2018	MW898020	India	
		CR-4	2018	MW898021	Bangladesh	
		CR-5	2018	MW898022	Bangladesh	
		CR-6	2018	MW898023	India	
		CR-7	2018	MW898024	Sri Lanka	
2022	Canada	1	2021	Unknown	India	Posso-De Los Rios CJ, et al. (28)
		2	2021		India	
		3	2021		India	
		4	2021		India	
		5	2021		India	
		6	2021		India	
		7	2021		India	
		8	2021		India	
2022	Vietnam	No_10_ITS1	2020	OM108103	Vietnam	Ngo TMC, et al. (29)
2022	Poland	600355/19	2019	OM951134	India	Uhrlaß S, et al. (15)
		600352/19	2019	OM951136	India	
		600356/19	2019	OM951138	India	
		600358/19	2019	OM951141	India	
		600354/19	2019	OM951142	India	
		600362/19	2019	OM951147	India	
2022	Austria	209287/21	2021	OM951140	India	Uhrlaß S, et al. (15)
2022	United Arab Emirates	600004/21	2020	OM951020	India	Uhrlaß S, et al. (15)
		600006/21	2020	OM951135	India	
2022	Switzerland	600269/19	2019	OM951144	Unknown	Uhrlaß S, et al. (15)
2022	Estonia	600280/19	2019	OM951139	Unknown	Uhrlaß S, et al. (15)
2022	Denmark	Danish-TINDO-isolate	2019	OM281735	Unknown	Astvad KMT, et al. (26)
2022	Greece	UOA/HCP F16949		ON182016	Unknown	Unpublished
2021	France	RES-AVC92	2021	OK632117	Unknown	Unpublished
		RES-BCH16		OK632122		
		RES-BCL120		OK632167		
		RES-HMD38		OK632190		
		RES-PSL40		OK632225		
		RES-PSL41		OK632226		
		RES-BCL75		OK632142		
2021	Switzerland	14070062	2014	MT858945	India(3), Bangladesh (1), Europe(3), Unknown(4)	Klinger M, et al. (21)
		16030031	2016	MT858946		
		16031162	2016	MT858947		
		17030525	2017	MT858948		
		17060294	2017	MT858949		
		17110903	2017	MT858950		
		18090688	2018	MT858951		
		19030383	2019	MT858952		
		19040978	2019	MT858953		
		19050687	2019	MT858954		
		19060507	2019	MT858955		
2021	Greece	AUH1273	2018	MW752105	Greek	Siopi M, et al. (22)
		AUH1357	2018	MW752107	Greek	
		AUH1665	2019	MW752108	Greek	
		AUH1678	2019	MW752109	Iran	
		AUH1598	2019	MW752110	Greek	
		AUH1621	2019	MW752111	Syria	
		AUH1650	2019	MW752111	Greek	
		AUH1687	2019	MW752112	Greek	
		AUH1745	2019	MW752113	Greek	
2020	Germany	208737/19	2019	MT328783	India	Nenoff P, et al. (10)
		211564/18	2018	MT330248	India	
		216377/17	2017	MT330249	Unknown	
		211542/18	2018	MT330250	Unknown	
		901538/18	2018	MT330251	Unknown	
		214677/16	2016	MT330252	India	
		218160/18	2018	MT330253	India	
		218691/18	2018	MT330254	Unknown	
		218360/18	2018	MT330255	Unknown	
		218676/19	2019	MT330256	Unknown	
		209934/19	2019	MT330278	India	
		600174/19	2019	MT330279	India	
		203513/19	2019	MT330280	Pakistan	
		205666/19	2019	MT330281	Iraq	
		600231/19	2019	MT330282	Unknown	
		205667/19	2019	MT330283	Iraq	
		202953/19	2019	MT330284	Libya	
		200618/19	2019	MT330285	Bahrain	
		217201/19	2019	MT330286	India	
		220575/19	2019	MT330287	Bangladesh	
		216532/19	2019	MT330288	India	
		214174/19	2019	MT330289	Germany	
		219238/19	2019	MT330290	Pakistan	
		600380/19	2019	MT330291	India	
		101549/20	2020	MT333225	Unknown	
		900138/20	2020	MT333226	Unknown	
		600002/20	2020	MT333227	Germany	
		200874/20	2020	MT333228	Unknown	
		204532/20	2020	MT333242	Bangladesh	
2020	Japan	CBS146623\NUBS19006	2019	LC508024	Nepal	Kano R, et al. (7)
		CBS146624\NUBS19007	2018	LC508728	India	
2010	Oman	WM10.87	2010	HQ014707	Oman	Unpublished
2008	Australia	05-297-2783	2007	EU200375	Unknown	Kong F, et al. (13)
		03-073-2580		EU200376	Unknown	

## Discussion

We confirmed the diagnosis of tinea corporis, tinea cruris and tinea faciei caused by *T. indotineae* (*T. mentagrophytes* ITS genotype VIII) in two Indian patients admitted to our outpatient clinic from clinical, etiological, and evolutionary taxonomic perspectives. To the best of our knowledge, this is the first report of *T. indotineae* from China. This pathogen was introduced as *T. indotineae* (7) in order to avoid confusion, as the species was referred to as *T. mentagrophytes* or *T. interdigitale* in earlier publications (15, 20). The species causes a human-to-human epidemic and has a high degree of drug resistance. Essential questions concern the mode and vectors of distribution, the species’ ultimate origin and transmission route, and the susceptibility profile of the wild-type population of the pathogen.

The novel emerging pathogen *T. indotineae* was found for the first time in the Guiyang area of southwest China. Our hospital is the largest hospital in Guizhou Province, which treats cases from all over the province. We investigated the dermatophytes collected in our department during the past five years using phenotypic and molecular characters, and found that *T. rubrum* still dominated (239/305, 77.9%) (unpublished data), which was consistent with epidemiological data published in China and elsewhere (39). In our collection, 31 clinical isolates (10.2%) were identified as members of the *T. mentagrophytes* complex (16). At present, this complex includes a large number of ITS genotypes (9); our clinical strains represent four of these. Sixteen strains belonged to *T. interdigitale* (genotypes I and II), which is mainly reported from pedal onychomycosis (11); our strains were from nail (9/16, 56.2%), face (5/16), scalp (1/16), and groin (1/16). Others belonged to *T. mentagrophytes* types VII, and IX. Two strains proved to be *T. indotineae* (**Figure 3**). This species has spread on a worldwide scale since 2007 (Our data, **Figure 8**). The earliest isolation of this species in the Guiyang area was in 2018, ten years after its initial discovery in Australia. The fungus emerged strongly in India, probably promoted by the inappropriate use of antifungals with corticosteroids which were sold over the counter without prescription (40). Both Chinese isolates were also from Indian patients, while local Chinese patients had no dermatophytosis caused by this species. The fungus thus seems to have been imported from India. The fact that the father in India of one of our patients had a similar rash concomitantly, even though the patients lived separately for years, suggests that *T. indotineae* may remain subclinical before becoming fulminant (10, 15). Interestingly, the two Indian patients denied a preceding history of dermatophytosis, and both lived in Guiyang for 3 years before the onset of disease. During the period they did not travel to India, nor had any history of travel to other provinces in China, and neither had contact with similar patients or animals. Dermatophytes may survive after physical contact in the form of spores at the body surface, provoking mild or no clinical symptoms. It is as yet unknown which factors led to proliferation after latency.

The imported pathogen has probably existed in Guiyang area for nearly 5 years, without further dissemination among the local population. Similar observations can be made in several countries other than India and Iran, such as Japan (7, 19), Canada (28) and France (24, 25), where the pathogen was first isolated around 2018, but there was no report of the pathogen spreading among the local population so far. Also in Australia, the earliest country where ‘genotype VIII’ was observed in 2007 (13), there has been no outbreak of this pathogen. It should be noted that the articles published so far are inconsistent with the time of strain collection (**Table 2**, **Figure 8**). Since 2017, sporadic cases have been reported in multiple countries in Europe (10, 20–22, 25), Canada (28), and Asia (19, 23, 29), but patients have mostly been migrants from the Indian subcontinent and neighbouring countries. For example, the 13 cases reported in France in 2022 were mainly from India and Bangladesh (24, 25); the two patients reported in Japan in 2020 were from India and Nepal, respectively (7) (**Table 2**). Therefore, we may speculate whether *T. indotineae* is associated with people of South Asian ancestry or with this genetic background, while other races show less susceptibility to this infection.

There is limited research investigating possible links between genetic polymorphism in key immune genes, human ancestry and gender, and susceptibility toward fungal infection. Genetic variants that lead to immunological susceptibility to fungi have been recognized (41). For example, endemic dimorphic fungi infection in North America by *Histoplasma, Coccidioides* and *Blastomyces* is observed in otherwise healthy individuals, but with a certain predilection for people of African, Native American, or Asian ancestry (41, 42). Infection with *Coccidioides immitis* among Filipino ancestry was found to be more common than in non-white persons (43). The rate of tinea imbricata, a tropical dermatophytosis with a characteristic pattern of skin lesions caused by *T. concentricum* (44), differs between people of different racial backgrounds despite similar exposure risk (44).

A thorough understanding of the correlation between *T. indotineae* and the genetic background of the host will help to clarify the taxonomic relationship of different genotypes in the *T. mentagrophyte*s complex and enhance the understanding of this emerging pathogen. If more data and genetic evidence are available to further confirm host susceptibility, clonal expansion of genotype VIII populations would be the best template for controlled studies. As opposed to genotype I and II host populations from all over the world, genotype VIII has not evolved to accommodate the cuticle of all races. In fact, previous studies have confirmed that *T. mentagrophyte*s and *T. interdigitale* are clonal offshoots of a single species (38). Our study also showed that these two species do not form monophyletic clades either and that the genotypes assigned between them are confused (10, 15). In particular, *T. interdigitale* type I and II representing *T. interdigitale* (including type strain CBS 428.63), *T. mentagrophyte*s types III, type III* representing *T. mentagrophyte*s (including type strain IHEM4268) and *T. mentagrophyte*s type VIII (the new species *T. indotineae*, with type strain CBS 146623) formed a clade with high support (ML/BI 81/0.97) (**Figure 3**), indicating that the relationship between this complex taxon still needs to be further clarified. We still do not have sufficient basis to confirm that genotype VIII is of zoophilic origin, similar to *T. mentagrophytes*.

Similar to previous studies (38, 45), our work also supports the possibility that *T. mentagrophytes* and *T. interdigitale* might be conspecific due to the lack of association between origin, strain morphology, genotype, and clinical manifestations. The strains belong to *T. interdigitale* types I and II mainly invaded nails (9/16) and faces (5/16), leading to anthropophilic biological behaviors with very mild inflammation. However, there were exceptions. JYP21091 of this cluster presented with highly inflammatory tinea capitis in an 8-year-old male child with a history of exposure to cats and rabbits, who remained scarred and alopecia after 6 months of standardized oral itraconazole treatment. Strains JYP22149 and JYP18108 (2/10, 20%) belonging to the ITS type IX cluster of *T. mentagrophytes* were isolated from adult toenails. In particular, the three isolates of *T. mentagrophytes* in ITS type VII range were from a family of three from a rabbit farm. Interestingly, the inflammatory response was completely different after infection with the same source of pathogen. The parents presented with clinically mild and self-limited tinea capitis, while the 5-year-old son (JYP18149=JYP 18-3) presented with highly inflammatory tinea capitis (30). All the above suggest that the traditional definitions of “anthropophilic”, “zoophilic” and “geophilic” corresponding to the clinical characteristics of “mild inflammation” and “severe inflammation” are not accurate and have their limitations, especially when used as the standard to define the boundary of fungal species. It is suggested to apply this concept only in an evolutionary sense.

In conclusion, our case report of *T. indotineae* from southwest China is indeed similar to other cases around the world, and the clinical, etiological, physiological, and drug resistance profiles are consistent with the pathogenic characteristics of this genotype, and itraconazole should be considered for treating recalcitrant cases. Meanwhile, the host and epidemiological characteristics provide us with a new perspective. Whether the emergence is limited to India is due to host factors or to local public health conditions remains questionable. Finally, the secondary drug resistance of many dermatophytes, including *Trichophyton indotineae* and *T. rubrum*, promoted by erratic use of antifungal drugs, needs attention.

## Data availability statement

The data presented in the study are deposited in the NCBI repository under the accession numbers OP961393-OP961423 for the new sequence of ITS and OQ054983 and OQ054984 for SQLE.

## Ethics statement

Written informed consent was obtained from the individual(s) for the publication of any potentially identifiable images or data included in this article.

## Author contributions

SJ and XL carried out the literature search and participated in the data analysis and drafted the manuscript. YaJ designed this project, phylogenetic tree construction, participated in the data analysis and revised the manuscript. WH and JZ carried out the statistical analysis. SA, GH, WL and YiJ participated in the design of the study and contributed to the discussion and revision of the manuscript. All authors contributed to the article and approved the submitted version.
