# Supplementary material for: The epidemic of the multiresistant dermatophyte Trichophyton indotineae has reached China
Source: Front Immunol. 2023 Feb 16;13:1113065. doi: 10.3389/fimmu.2022.1113065 (PMC9978415; doi:10.3389/fimmu.2022.1113065)
Supplement: Supplementary file 1 [file Table_1.pdf]

Table S-1. Information of Strains

number	locality	host	host nationality	sourse	ITS Genotype	Depositer	Genbank accession numbers of ITS
JYP18015	Guizhou, China	human	China	face	*T._interdigitale* I/II	Yanpin Jiang	OP961394
JYP18016			China	face	*T._interdigitale* I/II	Yanpin Jiang	OP961395
JYP18027			China	face	*T._interdigitale* I/II	Yanpin Jiang	OP961396
JYP20080			China	fingernail	*T._interdigitale* I/II	Yanpin Jiang	OP961407
JYP21005			China	toenail	*T._interdigitale* I/II	Yanpin Jiang	OP961410
JYP21020			China	toenail	*T._interdigitale* I/II	Yanpin Jiang	OP961411
JYP21029			China	toenail	*T._interdigitale* I/II	Yanpin Jiang	OP961412
JYP21066			China	face	*T._interdigitale* I/II	Yanpin Jiang	OP961413
JYP21070			China	toenail	*T._interdigitale* I/II	Yanpin Jiang	OP961414
JYP21071			China	face	*T._interdigitale* I/II	Yanpin Jiang	OP961415
JYP21091			China	head	*T._interdigitale* I/II	Yanpin Jiang	OP961416
JYP21097			China	toenail	*T._interdigitale* I/II	Yanpin Jiang	OP961417
JYP21100			China	groin, body	*T._interdigitale* I/II	Yanpin Jiang	OP961418
JYP21117			China	foot, toenail	*T._interdigitale* I/II	Yanpin Jiang	OP961419
JYP21300			China	toenail	*T._interdigitale* I/II	Yanpin Jiang	OP961420
JYP22108			China	toenail	*T._interdigitale* I/II	Yanpin Jiang	OP961422
JYP18047			China	face	*T._mentagrophytes* IX	Yanpin Jiang	OP961397
JYP18100			China	groin	*T._mentagrophytes* IX	Yanpin Jiang	OP961398
JYP18108			China	toenail	*T._mentagrophytes* IX	Yanpin Jiang	OP961399
JYP18133			China	unkonwn	*T._mentagrophytes* IX	Yanpin Jiang	OP961400
JYP18150			China	head	*T._mentagrophytes* IX	Yanpin Jiang	OP961402
JYP19047			China	head	*T._mentagrophytes* IX	Yanpin Jiang	OP961405
JYP20022			China	head	*T._mentagrophytes* IX	Yanpin Jiang	OP961406
JYP2022A			China	pus	*T._mentagrophytes* IX	Yanpin Jiang	OP961408
JYP2022B			China	hair	*T._mentagrophytes* IX	Yanpin Jiang	OP961409
JYP22149			China	toenail	*T._mentagrophytes* IX	Yanpin Jiang	OP961423
JYP18149			China	head, body	*T._mentagrophytes* VII	Yanpin Jiang	OP961401
JYP18152			China	body	*T._mentagrophytes* VII	Yanpin Jiang	OP961403
JYP18153			China	body	*T._mentagrophytes* VII	Yanpin Jiang	OP961404
JYP18010			India	groin	*T._mentagrophytes* VIII	Yanpin Jiang	OP961393
JYP22048			India	face, body	*T._mentagrophytes* VIII	Yanpin Jiang	OP961421
